# Investigating the long-term impact of a programme of mindfulness combined with exercise delivered online (MOVE) on individuals living with chronic pain-an exploratory one-year follow-up of a feasibility randomised control trial

**DOI:** 10.1371/journal.pone.0323508

**Published:** 2025-09-30

**Authors:** Orla Deegan, Mai Yoshitani, Brona Fullen, Maria Gordon, Orla McDonagh, Orlaith Sheerin, Jane Murphy, Oreluwa Oyebode, Ricardo Segurado, Conor Hearty, Maire Brid Casey, Catherine Doody

**Affiliations:** 1 Department of Rehabilitation Sciences, College of Health Sciences, QU Health Sector, Qatar University, Doha, Qatar; 2 School of Public Health, Physiotherapy and Sports Science, University College Dublin, Dublin, Ireland; 3 Clinical Development, Clinical Development Centre, Asahi Kasei Pharma Corporation, Tokyo, Japan; 4 Centre for Translational Pain Research, University College Dublin, Dublin, Ireland; 5 Department of Pain Medicine, Mater Misericordiae University Hospital, Dublin, Ireland; 6 Discipline of Physiotherapy, School of Medicine, Trinity College Dublin, Dublin, Ireland; Qatar University College of Nursing, QATAR

## Abstract

**Background:**

Online pain management programmes have evidence as effective interventions for individuals with chronic pain. This study was a one-year exploratory follow-up of a parallel group feasibility randomised controlled trial (RCT) investigating the effectiveness of participating in an online pain management programme that combined Mindfulness-Based Stress Reduction (MBSR) and exercise delivered online for individuals living with chronic pain (MOVE).

**Methods:**

Ninety-six individuals with chronic pain participated in an eight-week feasibility RCT comparing MBSR and exercise to an online self-management guide. Fourteen patient reported outcome measures (PROMS) were collected at baseline, post-intervention, three months post-intervention and at a one-year follow-up. Analysis of changes in PROMs between baseline and one-year follow-up in the current study were analysed using Student’s t-tests, and in addition, data were analysed using linear mixed models, examining effect sizes.

**Results:**

Forty-eight participants completed PROMs at a one-year follow-up. Small between group differences were observed in depression and the mental health component of the health-related quality of life score, in favour of the combined MBSR and exercise group. A further nine PROMS showed improvements in favour of the self-management group (SM). In the MOVE group, one-year follow-up scores demonstrated trends towards improvement compared to baseline scores for 10/14 PROMs. In the SM group positive changes were found in 13/14 PROMs. At one-year follow-up, results from the Patients Global Impression of Change Scale, demonstrated that 63.3% of participants in the combined mindfulness and exercise group, and 44.4% of participants in the self-management group reported improvement with a noticeable change.

**Conclusions:**

The results of this long-term exploratory study suggest no significant difference between groups for the investigated PROMs at one-year follow-up. A fully powered RCT examining the effectiveness of MBSR combined with exercise delivered in a live synchronous online format is warranted, to explore the factors to optimise long-term outcomes for people living with chronic pain.

## 1. Introduction

Chronic pain is defined by the International Association for the Study of Pain as “pain that persists or recurs for longer than three months” [1]. Globally, it has been estimated that 20% of adults suffer from pain and that another 10% of adults are diagnosed with chronic pain each year [2]. In Ireland, approximately 36% of Irish adults are affected by chronic pain which has been found to be associated with anxiety, depression, and sleep disturbances [3]. Chronic pain additionally poses a significant financial burden, contributing to increased healthcare costs and highlighting its impact as both a public health priority and a socioeconomic challenge [4].

Pain management programmes (PMP) are recommended as a valuable component in the management of chronic pain [5]. However, several obstacles to the implementation of PMPs have been identified, posing challenges to their widespread use. For example, geographical distance from PMP clinical settings, physical disabilities that limit mobility, and economic constraints present challenges for regular attendance at PMPs [6]. The online delivery of interventions for managing pain has the potential to overcome some of these barriers, providing more accessible and inclusive solutions for individuals living with chronic pain [7].

There is a growing body of evidence that supports exercise as a treatment modality for chronic pain. A Cochrane review conducted by Geneen and colleagues [4] noted that exercise for individuals with chronic pain may result in a reduction in pain severity and improvements in physical function; however, the authors reported that the overall quality of the evidence is low and further studies with more participants, longer interventions and follow-up periods are needed. Furthermore, Mindfulness-Based Stress Reduction (MBSR), a group-based third wave mindfulness-based intervention has an extensive evidence base in the treatment of chronic disease, including chronic pain [8], with studies reporting reduced pain catastrophising, increased self-efficacy and greater acceptance of chronic pain [9]. A recent systematic review [10] (n = 17 studies) investigated the effects of mindfulness-based interventions on a number of measures of health (i.e., physical functioning, disability, quality of life, pain). Nine studies included in the review investigated the effects of mindfulness-based eHealth interventions on pain, with four of these studies reporting superior effectiveness of the mindfulness-based intervention over a control group and five studies showing equal effectiveness to comparable interventions.

Previous research [11] highlights the challenges of encouraging individuals with chronic pain to increase their activity levels, suggesting these challenges were largely due to difficulties with exercise adherence and managing activity pacing. It is proposed that during participation in an MBSR programme, when factors such as reduced pain catastrophising and enhanced self-efficacy are facilitated, individuals may become more receptive to incorporating physical activity into their daily lives. Limited studies [12,13] have examined combining MBSR and exercise, and no studies to date have explored the long-term effects of delivering such an integrated intervention online.

This study was a one-year exploratory follow-up of the MOVE-Online trial, which investigated the acceptability and feasibility of an online MBSR and exercise programme for patients with chronic pain compared to an online self-management guide. In addition, the feasibility randomised control trial (RCT) investigated the effectiveness of the intervention using a number of patient-reported outcome measures (PROM) at baseline, post-intervention and at a three-month follow-up [14]. The aim of the current study was to conduct an exploratory investigation into the longer-term effectiveness of the combined MBSR and exercise intervention and the self-management (SM) intervention at a one-year follow-up.

## 2. Materials and methods

### 2.1. Study design

This study was a one-year exploratory follow-up study of a parallel-group feasibility RCT. The trial was registered at clincaltrials.gov (NCT04899622) and the trial protocol and RCT, results including a detailed methodology, have been published [14,15]. This study was approved by the Mater Misericordiae University Hospital Institutional Review Board (1/378/2124) and the University College Dublin Human Research Ethics Committee (LS-20–76-Deegan-Doody). The results of the current study are reported in accordance with the CONSORT guidelines [16].

### 2.2. Study sample size and study setting

A total of 96 participants were randomised to participate in the feasibility RCT, exceeding the target sample size. A sample size of 60 participants was considered appropriate for a feasibility study to assist with estimation of sample size for a fully powered RCT [17]. Each participant was allocated a unique code and randomised using the online software application ‘www.sealedenvelope.com’ by the primary researcher and the randomisation allocations were shared with the therapists implementing the intervention. It was not possible to blind the therapists or study participants to group allocation. Participants were be contacted by email by the primary researcher and informed of their group allocation.

The trial was a collaboration between University College Dublin, Ireland, and a pain management clinic in a large Irish university teaching hospital. The participants were based in Ireland and participated in the online intervention remotely, from their homes. Study recruitment began on February 1, 2021, and concluded on March 1, 2022.

### 2.3. Eligibility criteria

Adults aged 18 and older, diagnosed with any chronic pain condition lasting more than 12 weeks by either a pain medicine consultant or a general practitioner, and reporting a pain intensity greater than three on the Numerical Pain Rating Scale, were included in the study.

**Fig 1:**
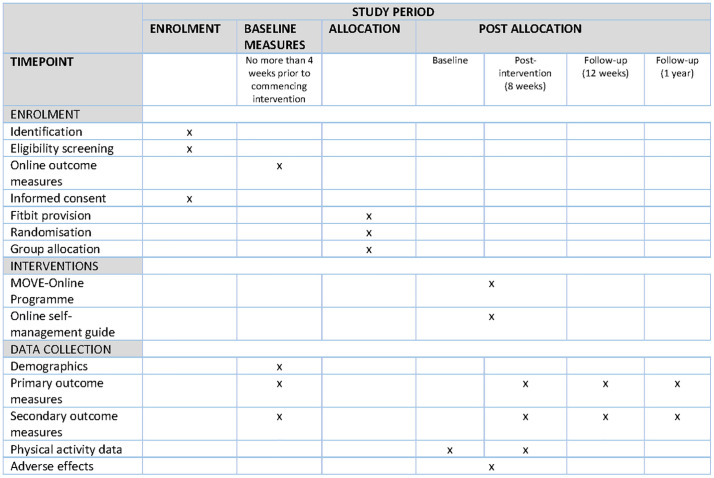
Standard Protocol Items: Recommendations for Interventional Trials (SPIRIT) Diagram.

Participants were required to be able to provide informed written consent and communicate effectively in the English language. The SPIRIT diagram (Fig 1) outlines the phases of the trial and data collection time points [18].

Exclusion criteria included; a requirement for further diagnostic evaluation (determined by a physician), presence of any contraindications to participation in an exercise programme (severe shortness of breath at rest, angina, uncontrolled diabetes or epilepsy, recent (previous three-weeks) myocardial infarction, pulmonary embolism, deep vein thrombosis, or asthma attack), presence of active cancer, coexisting participation (or in the previous three months) with any form of psychological, physiotherapy or supervised exercise, presence of substance misuse (diagnosed by physician), acute depression, suicidality, untreated psychosis and inability to participate in an online exercise programme. Other standard MBSR potential exclusion criteria, e.g., Post-Traumatic Stress Disorder, were investigated as exclusion criteria on an individual basis.

### 2.4. Data collection

Participants of the previously published feasibility RCT were invited to take part in a one-year follow-up study exploring the long-term effects of the online pain management programmes using the same battery of PROMs collected post-intervention and at a three-month follow-up. The PROMS were collected via online self-report questionnaires using Google Forms one year following completion of the programme. All participants were sent a link to the one-year follow-up online questionnaires via email. Follow-up reminder emails were sent, seven and 14 days after the initial email. The questionnaires were de-identified after collection. The de-identified data were transferred from Google Forms to Microsoft Excel, and subsequently to SPSS for data analysis.

**Fig 2:**
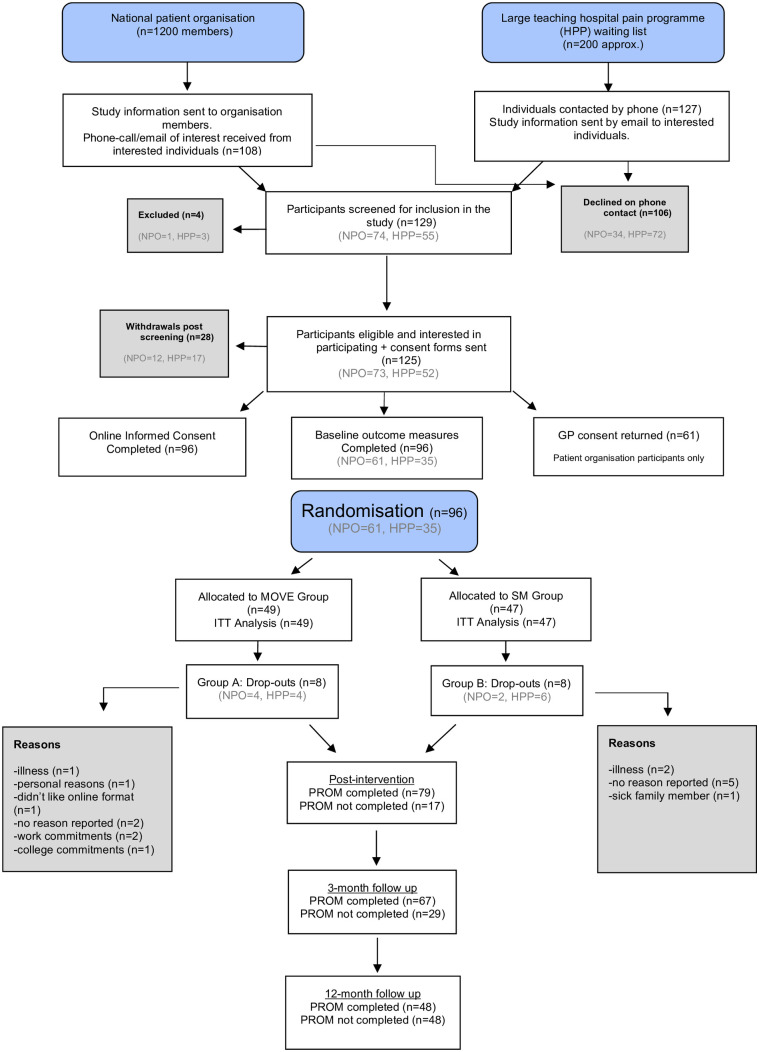
CONSORT diagram for feasibility studies. The diagram includes all of the outcomes pertaining to (i) Recruitment and Eligibility (ii) Data Collection and (iii) Attrition.

An adapted Consolidated Standards of Reporting Trials diagram (Fig 2) illustrates the participant flow through the study including initial telephone screening, recording of consent, collection of baseline measures via the online forms and randomisation to the MOVE group or the SM group. No items were missing from collected PROMs.

### 2.5. Procedure

#### Intervention A: MOVE online programme.

The interventions are described in detail by Deegan and colleagues [15] and are summarised below.

The intervention group participated in an eight-week online interactive intervention combining MBSR and exercise to assist in the management of chronic pain. The programme involved weekly online classes consisting of two hours of MBSR and one hour of exercise. The classes were delivered in a group setting and guided by Mindfulness Instructors and Physiotherapists. The mindfulness programme was tailored towards people with chronic pain and was based on the standard MBSR curriculum developed at The Centre for Mindfulness in Medicine, University of Massachusetts [19]. Participants were also encouraged to complete weekly home practices. Audio and video MBSR resources were provided via the study website. The exercise component included a one-hour supervised online exercise class delivered by a Physiotherapist. The class consisted of flexibility and strengthening exercises that progressed every two weeks. Exercise prescription of repetitions and sets were provided, and participants were encouraged to modify exercises as required. The exercises were also available in video format through the mobile application Physitrack.

#### Intervention B: Self-management guide.

The control group received access to an online chronic pain self-management guide. All participants received standard evidence-based self-help information accessible via the study website. The information included links to online resources, audio files and videos. The information was updated biweekly during the eight-week programme and participants received an email update each time the information was updated.

### 2.6. Patient reported outcome measures (PROM)

The PROMs included in the study are based on the IMMPACT (Initiative on Methods, Measurement and Pain Assessment in Clinical Trials) recommendations for outcome measures for use in chronic pain clinical trials [20]. Table 1 lists the validated PROMs which were completed by all participants, via the online forms. Full details of the outcome measures are provided in the study protocol [14]. A short description of the measures is provided below.

**Table 1:** Overview of the patient reported outcome measures collected at baseline, post-intervention, 12-week follow-up and one-year follow-up.

Patient Reported Outcome Measures	Instrument
Pain	Brief Pain Inventory (BPI) *Subscales: BPI Interference, BPI Composite Severity*
General and domain specific pain related disability	Pain Disability Index (PDI)
Perceived Improvement	Patient Global Impression of Change (PGIC) scale
Depression	Patient Health Questionnaire-9 (PHQ-9)
Health related quality of life	Short Form-36 Health Survey (SF-36) *Subscales: SF-36 Physical Component Score (PCS), SF-36 Mental Component Score (MCS)*
Anxiety	General Anxiety Disorder-7 (GAD-7)
Self-efficacy	Pain Self Efficacy Questionnaire (PSEQ)
Catastrophising	Pain Catastrophising Scale (PCS) *Subscales: PCS Rumination, PCS Helplessness, PCS Magnification)*
Fear of pain and consequent avoidance of physical activity	Fear Avoidance Belief Questionnaire (FABQ) *Subscales: FABQ Work, FABQ Physical Activity (PA)*
Participants gender, age, occupation, relationship status, level of education, pain diagnosis, years with chronic pain	Baseline online questionnaire

### 2.7. Outcome measure

The Brief Pain Inventory (BPI) was used to measure pain intensity and interference [21], and general and domain-specific pain-related disability was measured using the Pain Disability Index (PDI) [22]. The Patient Global Impression of Change (PGIC) scale was utilised to evaluate the patient’s perceptions regarding their overall improvement or lack of improvement as a result of participation in the intervention [23]. In the PGIC scale, the patients rated their perception of change following the treatment on a seven-point scale ranging from ‘very much improved’ to ‘very much worse.’ The Patient Health Questionnaire (PHQ-9) measured symptoms of depression, with higher scores indicating worse symptoms of depression [24]. The SF-36 was used to measure health-related quality of life and included two subscales measuring both physical and mental components related to quality of life (physical component score (PCS) and mental component score (MCS)) [25]. The Generalised Anxiety Disorder-7 was used to measure generalised anxiety [26] and pain catastrophising was measured using the Pain Catastrophising Scale (PCS) [27], with higher scores in both scales indicating worse symptoms. The Fear Avoidance Belief Questionnaire (FABQ) measured how patients’ beliefs contribute to their disability and consists of two subscales investigating physical activity and work [28]. Self-efficacy was measured using the Pain Self-Efficacy Questionnaire (PSEQ) with higher scores indicating better function [29].

### 2.8. Data analysis

The data were analysed by investigating the completion rates for the PROMs and the number of missing data. The primary analysis followed the intention-to-treat principle, where all participants were included in the analysis in the groups to which they were originally assigned, regardless of adherence to the intervention. The study was not powered to detect statistically significant between-group differences in outcomes, however analyses of between-group differences at a one-year follow-up were carried out for descriptive purposes. Baseline characteristics for demographic and clinical data for the participants were reported using descriptive statistics. Data from the online PROMs were analysed using SPSS, Version 29 [30]. The distribution of each PROM at baseline and one-year follow-up was assessed using histograms and the Shapiro-Wilk test to determine whether the data were normally distributed. This informed the use of parametric methods for normally distributed data and consideration of non-parametric approaches where distributions deviated significantly from normality.

The difference between baseline and one-year follow-up scores in PROMs was calculated by subtracting the baseline score from the one-year follow-up score for each measure. The differences were presented as means and 95% confidence intervals and analysed with respect to minimal clinically important differences (MCID) from relevant literature. A positive difference in PDI, GAD-7, PCS, PHQ-9, BPI and FABQ was interpreted as an improvement and a negative difference was interpreted as a worsening of symptoms. A negative difference in SF-36 and PSEQ was interpreted as improved and positive difference as a worsening of symptoms. To gain initial insight, Student’s t-tests were conducted on the differences between baseline and one-year follow-up scores and pre-post difference scores were present as means and 95% confidence intervals. Participants with missing outcomes were not included in this analysis. In addition, linear mixed models were conducted in SPSS Version 29 [30] using the MIXED procedure to examine changes in PROMs across four time points: baseline, post-intervention, three months, and one-year follow-up. Separate models were fitted for each PROM. Fixed effects included time (categorical), group (intervention vs. control), and the group × time interaction to assess differential change between groups over time. A random intercept for participants was included to account for individual baseline differences and repeated measures. An unstructured covariance matrix was specified for the repeated measures to allow for unequal variances and correlations at different time points. Although linear mixed models can accommodate missing data under the assumption that data are missing at random, no outcome data were missing in this dataset, so no imputation or additional missing data handling procedures were necessary. All analyses adhered to the intention-to-treat principle, with participants analysed in the groups to which they were originally assigned. Between-group effect sizes (Cohen’s d) and 95% confidence intervals were calculated at each time point and interpreted using conventional thresholds (small = 0.2, medium = 0.5, large = 0.8) [31].

## 3. Results

### 3.1. Response rate

A total of 96 participants were randomised to participate in the MOVE-Online trial. With respect to response rates at one-year follow up, 50.0% (n = 48) completed the online PROMs with notably higher response rates found in the MOVE group (62.5%) compared to the SM group (37.5%). There were no missing items in the PROM data. Participants were required to complete all items on the online forms in order to submit their responses.

### 3.2. Participant baseline characteristics

Participant baseline demographics are shown in Table 2. The mean age of participants in the MOVE group was 51.14 years of age, with n = 38 (77.6%) females and n = 10 (20.4%) males. A similar mean age was found in the SM group, (49.9 years), with n = 41 (87.2%) females and n = 6 (12.8%) males. The majority of the participants reported completing secondary (28.0%), third level (30.2%) or higher education (26.0%), however, there was a high percentage of participants who were not working, n = 43 (44.8%). Chronic pain symptoms with a duration of greater than 10 years were reported by 58 participants (60.4%). Additionally, 51.1% of participants (n = 49) experienced pain in between two and five areas of the body, while 45.8% (n = 44), reported pain in more than five areas.

**Table 2:** Participant Baseline Characteristics for the MOVE group and SM group.

Participant Characteristic	MOVE Group (n = 49) n (%) or mean ± SD	SM Group (n = 47) n (%) or mean ± SD	Total (n = 96)
Age (years)	51.14 ± 12.0	49.9 ± 12.1	50.54 ± 12.0
Gender			
Male	10 (20.4%)	6 (12.8%)	16 (16.6%)
Female	38 (77.6%)	41 (87.2%)	79 (82.3%)
Not specified	1 (2.0%)	0 (0.0%)	1 (1.0%)
Highest level of education achieved			
Primary	4 (8.2%)	1 (2.1%)	5 (5.2%)
Secondary	12 (24.5%)	15 (31.9%)	27 (28.2%)
Third level	13 (26.5%)	16 (34.0%)	29 (30.2%)
Higher level (post graduate)	15 (30.6%)	10 (21.3%)	25 (26.0%)
Other	5 (10.2%)	5 (10.6%)	10 (10.4%)
Work status			
Not working	23 (46.9%)	20 (42.5%)	43 (44.8%)
Student	2 (4.1%)	2 (4.3%)	4 (4.2%)
Retired	9 (18.4%)	10 (21.3%)	19 (19.8%)
Working part-time	5 (10.2%)	10 (21.3%)	15 (15.6%)
Working full-time	10 (20.4%)	5 (10.6%)	15 (15.6%)
Duration of CP symptoms			
< 5 years	7 (14.3%)	10 (21.3%)	17 (17.7%)
5–10 years	11 (22.4%)	10 (21.3%)	21 (21.9%)
>10 years	31 (63.3%)	27 (57.4%)	58 (60.4%)
Number of pain areas			
1 area	2 (4.1%)	1 (2.0%)	3 (3.1%)
2-5 areas	26 (53.1%)	23 (48.9%)	49 (51.1%)
>5 areas	21 (42.9%)	23 (48.9%)	44 (45.8%)
Number of CP diagnosis			
1 diagnosis	24 (25.0%)	12 (12.5%)	36 (37.5%)
2 diagnoses	11 (11.5%)	10 (10.4%)	21 (21.9%)
>2 diagnoses	10 (10.4%)	20 (20.8%)	30 (31.3%)
No diagnosis given	4 (4.2%)	5 (5.2%)	9 (9.4%)
Diagnosis			
Fibromyalgia	17 (17.7%)	17 (17.7%)	34 (36.5%)
Chronic Low Back Pain	9 (9.4%)	11 (11.5%)	20 (20.8%)
Arthritis	8 (8.3%)	9 (9.4%)	17 (17.7%)
Migraines	3 (3.1%)	6 (6.3%)	9 (9.4%)

Legend: CP, chronic pain; MOVE Group, Online interactive programme of MBSR and exercise; SM Group. Online self-management guide group

### 3.3. Analysis of PROMs

S1 File outlines the differences in findings between baseline and one-year follow-up scores for the investigated PROMs. In the MOVE group, one-year follow-up scores demonstrated non-significant trends towards improvement for PCS (Total, Rumination, Magnification and Helplessness subscales), PHQ-9, BPI (Interference and Composite Severity subscales), SF-36 (PCS subscale) and FABQ (Work and Physical Activity subscales). A deterioration in scores was found at a one-year follow-up for four of the investigated PROMs; PSEQ, PDI, GAD-7 and SF-36 (MCS subscale). Improvements were seen in the SF-36 (PCS subscale) in both the MOVE group (Mean −3.77 SD 16.28) and the SM group (Mean −8.4 SD 12.63), which exceeded the suggested MCID of 2.62–4.69 suggested by Grönkvist and colleagues [32]. For the remainder of the investigated PROMs in the MOVE group, MCIDs suggested in the available literature were not reached. Following an intention to treat analyses, small within group effect sizes were reported in the MOVE group at a one-year follow-up for all PROMs (0.03–0.28) (Table 3).

**Table 3:** Intention to Treat Analysis for Patient Reported Outcome Measures (PROMs).

Outcome Measure (Scoring range)			Baseline	Post Intervention	Effect size*	12 week Follow up	Effect size*	1 year Follow up	Effect size*
**PSEQ**	MOVE Group	Mean(95% CI)	30.10 (26.53, 33.68)	32.07 (28.01, 36.12)		31.42 (26.57, 36.26)		26.98 (22.84, 31.11)	
(0-60)		Δ (95% CI)		− 1.96 (−4.80, 0.87)	0.07	−1.31 (−5.79, 3.17)	0.16	3.58 (−0.30, 7.45)	0.2
	SM Group	Mean(95% CI)	26.98 (23.3, 30.63)	30.92 (26.64, 35.20)		31.47 (25.76, 37.19)		28.99 (23.97, 34.01)	
		Δ (95% CI)		−3.94 (−7.04, −0.85)	0.28	−4.50 (−9.90, 0.91)	0.33	−2.48 (−7.24, 2.28)	0.21
	Group Difference			1.98(−2.22, 6.17)	0.03	3.18 (−3.84, 10.20)	0.03	6.05 (−0.06, 12.16)	0.25
**PDI**	MOVE Group	Mean(95% CI)	37.53 (32.77-42.29)	36.58 (31.75, 41.41)		36.12 (30.78, 41.45)		39.46 (33.83, 45.08)	
(0-60)		Δ (95% CI)		0.95 (−2.59, 4.49)	0.06	1.42 (−2.21, 5.04)	0.13	−2.04 (−7.72, 3.64)	0.1
	SM Group	Mean(95% CI)	39.94 (35.07 −44.80)	38.12 (33.03, 43.22)		37.33 (31.41, 43.26)		35.98 (29.02, 42.94)	
		Δ (95% CI)		1.81 (−2.04, 5.66)	0.1	2.60 (−1.79, 6.99)	0.22	4.07 (−2.88, 11.03)	0.32
	Group Difference			−0.86 (−6.09, 4.37)	0.11	−1.19 (−6.89, 4.51)	0.08	−6.12 (−15.09, 2.86)	0.35
**GAD-7**	MOVE Group	Mean(95% CI)	9.27 (7.59, 10.94)	7.53 (5.87, 9.18)		11.77 (7.01, 16.53)		10.19 (7.85, 12.53)	
(0-21)		Δ (95% CI)		1.74 (0.54, 2.95)	0.3	−2.50 (−7.18, 2.18)	0.13	−1.0 (−3.38, 1.37)	0.05
	SM Group	Mean(95% CI)	10.26 (8.55, 11.96)	9.24 (7.50, 10.98)		9.62 (3.71, 15.53)		9.89 (7.01, 12.78)	
		Δ (95% CI)		1.02 (−0.29, 2.32)	0.22	0.64 (−5.21, 6.48)	0.28	0.44 (−2.48, 3.36)	0.32
	Group Difference			0.73 (−1.05, 2.50)	0.25	−3.14 (−10.63, 4.35)	0.19	−1.44 (−5.10, 2.21)	0.35
**PCS (total)**	MOVE Group	Mean(95% CI)	20.14 (16.05, 24.24)	17.64 (13.50, 21.78)		18.56 (13.81, 23.30)		18.69 (14.09, 23.28)	
(0-52)		Δ (95% CI)		2.51 (−0.25, 5.26)	0.15	1.59 (−1.19, 4.37)	0.15	1.49 (−1.99, 4.97)	0.16
	SM Group	Mean(95% CI)	22.81 (18.63, 26.99)	20.52 (16.17, 24.87)		21.91 (16.76, 27.05)		20.01 (14.75, 25.35)	
		Δ (95% CI)		2.29 (−0.71, 5.29)	0.23	0.90(−2.43, 4.23)	0.26	2.72 (−1.53, 6.97)	0.37
	Group Difference			0.22 (−3.86, 4.29)	0.10	0.69 (−3.65, 5.02)	0.10	−1.74 (−6.70, 4.25)	0.64
**PCS (Rumination)**	MOVE Group	Mean(95% CI)	5.82 (4.41, 7.22)	5.33 (3.91, 6.76)		6.17 (4.52, 7.82)		5.49 (3.96, 7.02)	
(0-16)		Δ (95% CI)		0.48 (−0.59, 1.56)	0.07	−0.35 (−1.30, 0.60)	0.05	0.25 (−1.09, 1.57)	0.07
	SM Group	Mean(95% CI)	6.94 (5.50, 8.37)	6.06 (4.55, 7.56)		6.78 (4.98, 8.57)		5.12 (3.29, 6.94)	
		Δ (95% CI)		0.88 (−0.28, 2.04)	0.22	0.16 (−1.00, 1.32)	0.16	1.91 (0.28, 3.54)	0.49
	Group Difference			−0.40 (−1.98, 1.18)	0.08	−0.51 (−2.01, 0.99)	0.11	−1.66 (−3.77, 0.44)	0.29
**PCS (Magnification)**	MOVE Group	Mean(95% CI)	4.22 (3.25, 5.20)	3.62 (2.64, 4.61)		3.89 (2.18, 5.00)		4.37 (3.22, 5.53)	
(0-12)		Δ (95% CI)		0.60 (−0.10, 1.30)	0.16	0.33 (−0.41, 1.08)	0.16	−1.68 (−1.10, 0.76)	0.01
	SM Group	Mean(95% CI)	4.75 (3.75, 5.74)	4.48 (3.44, 5.52)		4.61 (3.38, 5.84)		4.95 (3.59, 6.32)	
		Δ (95% CI)		0.27 (−0.50, 1.03)	0.16	0.14 (−0.76, 1.04)	0.27	−0.87 (−1.34, 0.97)	0.20
	Group Difference			0.33 (−0.70, 1.37)	0.14	0.20 (−0.98, 1.37)	0.01	0.19 (−1.46, 1.50)	0.15
**PCS (Helplessness)**	MOVE Group	Mean(95% CI)	10.10(8.102, 12.10)	8.65 (6.60, 10.66)		7.55 (5.32, 9.78)		8.50 (6.23, 10.77)	
(0-24)		Δ (95% CI)		1.46 (−0.02, 2.93)	0.20	2.66(0.93, 4.18)	0.43	1.63 (−0.20, 3.45)	0.28
	SM Group	Mean(95% CI)	11.128 (9.09, 13.17)	9.68 (7.55, 11.80)		10.03 (7.55, 12.50)		10.47 (7.88, 13.09)	
		Δ (95% CI)		1.45 (−0.15, 3.05)	0.27	1.10 (−0.82, 3.02)	0.34	0.64 (1.57, 2.87)	0.35
	Group Difference			0.004 (−2.17, 2.18)	0.05	1.45 (−1.06, 3.97)	0.21	0.99 (−1.86, 3.84)	0.41
**PHQ-9**	MOVE Group	Mean(95% CI)	12.22 (10.24, 14.21)	10.19 (8.17, 12.21)		11.89 (9.75, 14.03)		11.50 (9.50, 13.50)	
(0-27)		Δ (95% CI)		2.04 (0.70, 3.38)	0.28	0.34 (−1.21, 1.88)	0.15	0.77 (−1.10, 2.64)	0.20
	SM Group	Mean(95% CI)	12.70 (10.67, 14.73)	12.09 (9.96, 14.20)		12.31 (9.93, 14.68)		12.67 (10.25, 15.10)	
		Δ (95% CI)		0.62 (−0.85, 2.08)	0.08	0.40 (−1.44, 2.23)	0.25	−0.01 (−2.29, 2.29)	0.08
	Group Difference			1.42 (−0.57, 3.41)	0.06	−0.06 (−2.46, 2.34)	0.21	0.78 (−2.17, 3.73)	0.13
**BPI (Interference)**	MOVE Group	Mean(95% CI)	5.34 (4.70, 5.98)	4.32 (3.60, 5.03)		4.57 (3.81, 5.34)		5.06 (4.31, 5.81)	
(0-10)		Δ (95% CI)		1.03 (0.40, 1.65)	0.4	0.77 (0.02, 1.52)	0.23	0.27 (−0.42, 0.96)	0.03
	SM Group	Mean(95% CI)	5.77 (5.12, 6.43)	5.11 (4.34, 5.87)		5.40 (4.49, 6.31)		5.28 (4.36, 6.20)	
		Δ (95% CI)		0.67 (−0.16, 1.35)	0.21	0.37 (−0.52, 1.26)	0.01	0.51 (−0.35, 1.36)	0.18
	Group Difference			0.36 (−0.57, 1.29)	0.41	0.40 (−0.76, 1.56)	0.44	−0.23 (−1.34, 0.87)	0.46
**BPI (Composite Severity)** (0-10)	MOVE Group	Mean(95% CI)	5.46 (4.94, 5.98)	5.28 (4.76, 5.80)		5.07 (4.42, 5.71)		5.29 (4.71, 5.87)	
		Δ (95% CI)		0.18 (−0.27, 0.62)	0.09	0.39 (−0.15, 0.94)	0.19	0.17 (−0.41, 0.75)	0.08
	SM Group	Mean(95% CI)	5.66 (5.13, 6.19)	5.63 (5.08, 6.18)		5.41 (4.66, 6.15)		5.08 (4.37, 5.80)	
		Δ (95% CI)		0.029 (−0.45, 0.51)	0.01	0.25 (−0.41, 0.91)	0.18	0.58 (−0.12, 1.29)	0.31
	Group Difference			0.145 (−0.51, 0.81)	0.21	0.14 (−0.72, 0.99)	0.12	−0.42 (−1.33, 0.50)	0.56
**FABQ (Work)**	MOVE Group	Mean(95% CI)	17.53 (13.83, 21.24)	18.36 (14.31, 22.40)		12.87 (8.46, 17.28)		17.39 (13.18, 21.61)	
(0-42)		Δ (95% CI)		0.82 (−3.30, 1.65)	0.07	4.66 (0.17, 9.15)	0.29	−0.09 (−3.84, 3.67)	0.13
	SM Group	Mean(95% CI)	17.98 (14.20, 21.76)	17.74 (13.51, 21.97)		13.65 (8.37, 18.92)		16.25 (11.13, 21.36)	
		Δ (95% CI)		0.24 (−2.46, 2.94)	0.07	4.33 (−1.02, 9.68)	0.32	1.96 (−2.70, 6.63)	0.12
	Group Difference			−1.06 (−4.73, 2.6)	0.11	0.33 (−6.66, 7.31)	0.002	−2.05 (−8.04, 3.95)	0.17
**FABQ (Physical**	MOVE Group	Mean(95% CI)	12.14 (10.23, 14.06)	11.30 (9.36, 12.24)		11.78 (9.73, 13.83)		12.2 (9.95, 14.45)	
**Activity)** (0–24)		Δ (95% CI)		0.84 (−0.89, 2.58)	0.1	0.37 (−1.70, 2.43)	0.09	−0.14 (−2.29, 2.01)	0.06
	SM Group	Mean(95% CI)	14.38 (12.87, 16.79)	11.84 (9.76, 13.91)		12.19 (9.77, 14.60)		12.03 (9.27, 14.79)	
		Δ (95% CI)		2.99 (1.12, 4.87)	0.54	2.64 (0.22, 5.07)	0.55	2.89 (0.24, 5.54)	0.38
	Group Difference			−2.15 (−4.71, 0.40)	0.02	−2.28 (−5.46, 0.91)	0.24	−3.03 (−6.44, 0.38)	0.09
**SF-36 (PCS)**	MOVE Group	Mean(95% CI)	35.55 (31.92, 39.18)	35.12 (31.2, 39.02)		35.65 (31.92, 39.39)		39.28 (33.77, 44.79)	
(0-100)		Δ (95% CI)		0.44 (−2.8, 3.68)	0.03	−0.10 (−3.03, 2.83)	0.02	−3.73 (−9.25, 1.80)	0.27
	SM Group	Mean(95% CI)	31.44 (27.73, 35.15)	35.97 (31.81, 40.13)		33.843 (29.57, 38.12)		39.86 (32.93, 46.79)	
		Δ (95% CI)		−4.53 (−8.04, −1.02)	0.24	−2.41 (−5.97, 1.16)	0.13	−8.43 (−15.33, −1.54)	0.62
	Group Difference			4.97 (0.20, 9.75)	0.08	2.30 (−2.31, 6.92)	0.35	4.71 (−4.13, 13.54)	0.01
**SF-36 (MCS)**	MOVE Group	Mean(95% CI)	47.05 (41.07, 53.04)	53.13 (46.69, 59.57)		50.19 (43.43, 56.96)		42.65 (34.70, 50.60)	
(0-100)		Δ (95% CI)		−6.07 (−12.11, −0.04)	0.31	−3.14 (−8.74, 2.46)	0.22	4.36 (−3.9, 12.62)	0.21
	SM Group	Mean(95% CI)	47.21 (41.10, 53.32)	49.31 (42.4, 56.23)		49.38 (41.54, 57.22)		39.66 (29.88, 49.43)	
		Δ (95% CI)		−2.10 (−8.62, 4.42)	0.01	−2.17 (−9.00, 4.67)	0.13	7.61 (−2.39, 17.61)	0.37
	Group Difference			−3.97 (−12.85, 4.91)	0.25	−0.97 (−9.81, 7.86)	0.18	−3.26 (−16.21, 9.70)	0.05

*Cohen d computed as mean difference relative to pooled standard deviations (baseline standard deviations used in both within and between-group calculations).

Δ indicates change from baseline; BPI, Brief Pain Inventory; FABQ, Fear Avoidance Belief Questionnaire; GAD-7, General Anxiety Disorder; MOVE group, online interactive programme of mindfulness-based stress reduction and exercise; MCS, Mental component Score; PCS, Pain Catastrophizing Scale; PHQ- 9, Patient Health Questionnaire; PDI, Pain Disability Index; PSEQ, Pain Self-Efficacy Questionnaire; SF-36, 36-Item Short Form Survey; SM group, online self-management guide.

In the SM group, trends towards improvements were found in the majority of the one-year follow-up scores, when compared to the baseline scores for the investigated PROMs, with the exception of SF-36 (MCS subscale). Notable within group improvements were seen in PCS (Rumination subscale) and FABQ (Work subscale), however, these measures did not reach the MCIDs suggested in the literature [33]. In the SM group, improvements reaching the MCIDs suggested in the literature were noted in the pre-post difference scores for PDI [34] and SF 36 (PCS subscale) [32]. Small to medium within group effect sizes were reported at a one-year follow up for the SM group (<0.2–0.62).

#### 3.4.1. Intention to treat analyses: Investigating between group differences.

Prior to analysis, model assumptions were assessed; residuals were found to be approximately normally distributed. Two of the investigated PROMs (PHQ-9, SF-36 (MCS subscale)), found between group differences in favour of the MOVE group, while in nine PROMs (PSEQ, PDI, GAD-7, PCS, BPI (Interference and Composite Severity subscales), FABQ (Work and Physical Activity subscales), and SF-36 (PCS subscale) between group differences were found in favour of the SM group. On examination of effect sizes, small (d < 0.2) to medium (d = 0.64) effect sizes were found across all PROMs and are highlighted in Table 3

### 3.4. Patient global impression of change

At a one-year follow up, a higher number of participants reported feeling improved with a noticeable change (‘minimally improved’, ‘much improved’ or ‘very much improved’) in the MOVE group (n = 19, 63.3%), when compared to the SM group (n = 8, 44.5%), reported in Table 4. Similar results were found immediately post interventions with 65.0% of participants (n = 28) in the MOVE group and 47.0% of participants (n = 17) in the SM group reporting feeling improved.

**Table 4:** Patient Global Impression of Change (PGIC).

PGIC Response	Post Intervention		12-week follow-up		1-year follow-up	
	MOVE Group	SM Group	MOVE Group	SM Group	MOVE Group	SM Group
*‘Since the start of the study, my general health is:’*	n (%)	n (%)	n (%)	n (%)	n (%)	n (%)
Very much worse	0 (0.0)	0 (0.0)	2 (4.9)	0 (0.0)	0 (0.0)	0 (0.0)
Much worse	0 (0.0)	2 (5.6)	1 (2.4)	1 (3.9)	5 (16.7)	2 (11.1)
Minimally worse	5 (11.6)	5 (13.9)	1 (2.4)	5 (19.2)	3 (10.0)	2 (11.1)
No change	10 (23.3)	12 (33.3)	13 (31.7)	13 (50.0)	3 (10.0)	6 (33.3)
Minimally improved	17 (39.5)	11 (25.6)	14 (3.2)	6 (23.1)	12 (40.0)	5 (27.8)
Much improved	11 (25.6)	4 (11.1)	10 (24.4)	1 (3.9)	6 (20.0)	3 (16.7)
Very much improved	0 (0.0)	2 (5.6)	0 (0.0)	0 (0.0)	1 (3.3)	0 (0.0)
**Total n (%)**	**43 (100)**	**36 (100)**	**41 (100)**	**26 (100)**	**30 (100)**	**18 (100)**

Legend: MOVE Group, Online interactive programme of MBSR and exercise; SM Group. Online self-management guide group; n, number of participants; %, percentage of participants; PGIC, Patient global impression of change.

### 3.6. Evaluation of participant characteristics by outcome measure completion at one-year follow-up

Descriptive data comparing the baseline socio-demographic and clinical characteristics of those who returned the outcome measures at one-year follow-up with those who did not are presented in S2 File and S3 File. The mean age of those who returned the outcome measures was slightly higher (52 years) compared to those who did not return the measures (48 years). Two recruitment sources were used to invite individuals to participate in the study. From the national patient organisation for individuals living with chronic pain, 62.3% of participants (n = 38) returned the one-year follow-up PROMS, compared to 28.6% of the participants (n = 10) recruited from the hospital waiting list for a pain management clinic. In terms of clinical characteristics, those who did not return the measures had marginally higher baselines scores, indicating worse symptoms than those who did return the PROMS, for measures of depression (PHQ-9), anxiety (GAD-7), pain catastrophising (PCS), and fear-avoidance behaviour (FABQ). For the mental component of the health-related quality of life measure (SF-36 MCS) those who did not return the measure had slightly lower scores which indicated worse symptoms at baseline than those who did return the measures.

## 4. Discussion

This study reports one-year follow-up data from the MOVE online trial; a study which investigated the acceptability and feasibility of an online MBSR and exercise programme for individuals living with chronic pain in comparison to an online self-management guide [14]. To our knowledge, this study is the first of its kind to investigate the long-term effectiveness of the synchronous online delivery of both MBSR and exercise for people living with chronic pain.

A total of 96 participants took part in the current study, exceeding the target sample size of 60 which was considered appropriate for the feasibility of a fully powered RCT [17]. Sixteen participants withdrew after group allocation (eight from the MOVE group and eight from the SM group). The overall response rate of the one-year follow-up was 50.0% (n = 48) with 62.5% of participants responding from the MOVE group and 37.5% from the SM group. A comparable response rate was found at a one-year follow-up of the ExACT trial, which similarly investigated a combined exercise and psychological (acceptance and commitment therapy) intervention for adults with chronic pain, and had an overall response rate at one-year of 47.4% [35]. Furthermore, when the baseline clinical characteristics of those who responded to the one-year follow-up in the ExACT trial were investigated, similar results to the current study were found, with participants who did not return the one-year PROMs also showing higher baseline scores for depression, anxiety and pain catastrophising.

A small number of studies investigating online pain management programmes have reported similar long-term follow-up data. Dear and colleagues [36] examined outcomes from an RCT investigating an internet-delivered pain management programme with both 12- and 24-month follow-up data. The trial reported completion rates of outcomes measures notably higher than those seen in the current study with data collected from 78.0% of participants at the 12-month follow-up and 79.0% of participants at the 24-month follow-up. Dear and colleagues reported that to encourage participants to complete the follow-up questionnaires, participants were sent emails and were contacted by phone on a number of occasions over a four-week period. In addition, study participants were also provided with written feedback about their symptom scores and responses to the questionnaires via email each time they completed the questionnaires. This feedback, in addition to the follow-up phone calls may have contributed to the increased response rate found in the current study and would be a consideration for the design of a future fully powered RCT investigating internet delivered pain management programmes. Collecting the PROM data via google forms in the current study was deemed to be an efficient method of data collection for the baseline measures and short-term follow-ups with PROMS, however, while no missing data was identified from the collected one-year data, the completion rates were low (50.0%). Additional strategies such as pre-contacting the participants, using other survey formats in conjunction with online surveys, and the use of phone call reminders were identified by Wu et al. (2022) as methods to help to increase the response rates after clinical interventions [37]. These methods are recommended for use in a larger RCTs, in particular with long-term follow-ups (one-year) which appear to report significantly lower response rates. In the current study, it is notable that the response rate was significantly higher for the MOVE group of participants compared to the SM group (62.3% versus 37.7%). The live online synchronous and interactive method of delivery in the MOVE group compared to the self-directed asynchronous method for the SM group may have influenced the larger response rate for the former, however further research is warranted to confirm this.

In the exploratory analysis of the within and between groups differences of PROMS at a one-year follow-up, it was noted that two of the between group differences were found to be in favour of the MOVE group (PHQ-9 and SF-36-MCS) and nine of the between group differences were in favour of the SM group (PSEQ, PDI, GAD-7, PCS, BPI (interference and composite severity subscales) FABQ (Work and Severity subscales) and SF-36-PCS), however, all but one of the between group differences were small, had small effect sizes and did not meet the MCID. Within group comparisons between baseline and one-year follow up scores show trends towards improvements in the majority of measures of the SM group (with the exception of SF-36 MCS), and all except three PROMs (PDI, PSEQ and GAD-7) in the MOVE group. The changes found in the current study were in general small when compared to MCIDs investigated in previous literature. For example, for the PSEQ, an investigation of patients with chronic low back pain reported MCIDs ranging from 5.5 to 8.5 out of 60 points [37], while in another study investigating chronic low back pain by [38] a change of 9–11 points on the PSEQ corresponded to the MCID. The mean change values in the current study were notably lower (MOVE group, Mean 2.5 SD 12.55; SM group, Mean-2.63 SD12.19) suggesting that the recommended MCID was not reached in the examined cohort. Changes at one-year follow-up in PCS (total) score were higher in the SM group (Mean 4.58, SD 12.37) compared to the MOVE group (Mean 2.28, SD 10.29). However, in a study by Monticone and colleagues [33] investigating the responsiveness and minimal important change of the PCS in individuals with chronic low back pain undergoing multidisciplinary rehabilitation, a MCID of 9 points (95% CI 6–10) was found for people with low pain catastrophising scores (PCS score <30) and 11 points (95% CI 8–14) for people with high pain catastrophising scores (PCS score >30), notably higher values than those identified in the current study (MOVE group, Mean 2.28 SD 10.29; SM group, Mean 4.58 SD 12.37). A study by Hawk and colleagues [34] investigated the PDI in older adults with chronic musculoskeletal pain and reported an MCID of 6 points. In the SM group the difference score from baseline to one-year follow-up for PDI reached the suggested MCID (Mean 6.26, SD 19.94). The changes in the health-related quality of life subscale, SF-36-PCS, suggested notable improvements in favour of the MOVE group with both the MOVE and SM group reaching the MCID as suggested by Grönkvist and colleagues (MCID 2.62 to 4.69 for SF-36 PCS) [32]. Grönkvist et al (2024) investigated a chronic pain population participating in an interdisciplinary pain rehabilitation intervention, allowing for more appropriate comparisons of MCIDs between this study and the current study. A similar investigation of a multidisciplinary pain management programme, with a six and 12-month follow-up, also investigated health related quality of life using the SF-36 outcome measure. While small improvements were found in the scores in this study, no statistically significant changes in the measures were found from immediately post intervention to a 12-month follow-up [39]. Whilst there appears to have been some effect within both groups, this did not manifest between groups to the magnitude it might have been expected and the self-management intervention appears to have conferred similar effects across the explored measures as the combined mindfulness and exercise intervention. It appears that a large number of empirical studies have been conducted to estimate MCID in chronic pain populations, but the studies differ considerably with regard to methodology, clinical conditions, and findings making comparisons between studies challenging. It has been suggested that baseline pain may likely influence measures of MCID [40], but it remains unclear whether other clinical or methodological factors cause variation.

A study by Nordstoga and colleagues [41] investigating low back pain, reported that multiple pain sites are negatively associated with prognosis and outcome in low back pain. In terms of characteristics of the current study participants, 45.8% of participants reported more than five pain areas at baseline. It has been suggested that findings such as a high number of pain areas and increased pain sensitivity in areas remote from the primary area of pain are highly suggestive of central sensitisation [42], which has been demonstrated in many chronic pain conditions [43,44]. Furthermore, psychological interventions, such as MBSR [45], Cognitive Behavioural Therapy [46] and Acceptance and Commitment Therapy [47] have reported positive effects on the pain management of individuals living with pain presenting with symptoms suggestive of central sensitisation. However, the majority of these studies examine the short-term effects of the pain management interventions, with few studies examining the effects of interventions over a longer period, in particular with participants with symptoms suggestive of central sensitisation. In the current study, the baseline duration of chronic pain for 60.4% of the participants was greater than 10 years, which is significantly higher than the national average in Ireland reported as 7.6 years in the PRIME study [3]. This long duration of pain symptoms may play a further role in the small improvements identified in PROMs. It has been suggested in previous literature investigating physiotherapy interventions for chronic low back pain that those who had symptoms for greater than six months experienced significantly less functional improvements than participants who had symptoms for less than six months [48].

Patients global impression of change is suggested to be an important outcome to consider when evaluating interventions for chronic pain [49]. In the current study, at a one-year follow up, a higher percentage of participants reported feeling improved, measured using the PGIC scale, in the MOVE group (n = 19: 63.3%), compared to the SM group (n = 8: 44.4%) (‘minimally improved’, ‘much improved’ or ‘very much improved’). It is not clear as to why this is the case, however the MOVE group had additional interaction with health professionals on a weekly basis during the eight-week programme and in addition had weekly peer interaction which may have provided a more impactful influence on participants perception of improvement over time. In the previously mentioned ExACT trial [35], a combined exercise and psychological intervention was also conducted and compared to a group participating in exercise only. It was also reported that a higher proportion of participants in a combined Exercise and ACT group reported feeling improved with noticeable changes in the PGIC outcome measure at a one-year follow-up.

The strengths of the current study include the examination of a broad diagnostic profile of patients with mixed pain conditions, which may be representative of populations who present for chronic pain treatment in hospital pain clinics. Additionally, the one-year follow-up time period is advantageous in investigating the long-term feasibility, acceptability of the interventions and to explore the long-term efficacy of the interventions which is important for patients with chronic conditions. The limitations discussed in the previous publication [15] reporting the post-intervention and 12-week follow-up results of this feasibility study are also applicable to this one-year follow-up. The limitations of this study include a lack of blinding of participants and clinicians which is similar to other chronic pain trials [50] and is usual for trials of complex interventions for chronic pain. In addition, financial barriers which may limit access to the devices necessary to engage with an online programme, as well as limits in individual’s digital literacy are significant barriers to participation in an online pain management programme. Several studies have highlighted strategies such as tablet loan programmes, identifying Wi-Fi hot spots and additional community outreach programmes to overcome the associated barriers with online treatment programmes [51]. A further limitation for this one-year follow-up study is the low response rate (50.0% overall). In addition, data was not collected on the extent to which participants continued to engage with treatment-related strategies following the intervention period, which limits our ability to assess the sustained use of techniques and their potential influence on outcomes at one-year follow-up.

## 5. Conclusion

The results of this long-term follow up feasibility trial suggest that both the online MOVE intervention group and the online SM guide group formats explored in this study are feasible and acceptable. The examined PROMS showed trends towards improvements across a large number of measures, including measures of pain, function and psychological health, with no notable between group differences identified in the explored sample. These modest within-group effects, and the limited between-group changes, are in line with expectations for a feasibility trial, which is not designed to detect efficacy but to assess the practicality and methodological soundness of conducting a larger trial. Despite the lack of statistically significant differences, several outcomes demonstrated favourable trends in the MOVE group, indicating a potential for clinical benefit that merits further investigation. Moreover, qualitative feedback [52] and engagement data highlighted the acceptability and value of the intervention, particularly in areas of pain management and self-efficacy, supporting its relevance and acceptability in an online format. A fully powered RCT examining the effectiveness of MBSR combined with exercise delivered in a live synchronous online format, with a focus on exploring the long-term impact of online pain management programmes would be recommended.

## Supporting information

S1 File Differences between baseline and one-year follow-up scores for the investigated PROMS. (DOCX)

S2 File Sociodemographic characteristics for participants who returned outcome measures at 1-year and those who did not. (DOCX)

S3 File Clinical characteristics for participants who returned outcome measures at 1-year and those who did not. (DOCX)
