# Supplementary material for: Investigating the long-term impact of a programme of mindfulness combined with exercise delivered online (MOVE) on individuals living with chronic pain-an exploratory one-year follow-up of a feasibility randomised control trial
Source: PLoS One. 2025 Sep 30;20(9):e0323508. doi: 10.1371/journal.pone.0323508 (PMC12483213; doi:10.1371/journal.pone.0323508)
Supplement: S1 File — (DOCX) [file pone.0323508.s001.docx]

**Supplementary File 1 (S1):** Differences between baseline and one-year follow-up scores for the investigated PROMS

|  | **MOVE Group** | | | | **SM Group** | | | |
| --- | --- | --- | --- | --- | --- | --- | --- | --- |
| **Variable** | **Baseline (n=49)** | **One-year (n=31)** | **Pre-post difference** | | **Baseline (n=47)** | **One-year (n=17)** | **Pre-post difference** | |
|  | **Mean (SD)** | **Mean (SD)** | **Mean (SD)** | **95% CI** | **Mean (SD)** | **Mean (SD)** | **Mean (SD)** | **95% CI** |
| **PSEQ** (0-60) | 29.34 (12.31) | 26.79 (14.07) | 2.55 (12.55) | -2.22, 7.32 | 27.79 (12.45) | 30.42 (11.10) | -2.63 (12.19) | -8.50, 3.24 |
| **PDI** (0-70) | 37.66 (17.32) | 39.14 (15.97) | -1.48 (15.53) | -7.39, 4.43 | 41.26 (16.14) | 35.00 (17.15) | 6.26 (19.94) | -3.35, 15.87 |
| **GAD-7** (0-21) | 8.97 (5.04) | 9.24 (6.46) | -0.28 (5.67) | -2.43, 1.88 | 9.89 (5.15) | 8.16 (4.43) | 1.74 (5.41) | -0.87, 4.35 |
| **PCS** (0-52)  (total) | 20.32 (13.29) | 18.07 (14.67) | 2.28 (10.29) | -1.64, 6.19 | 21.84 (12.08) | 17.26 (13.43) | 4.58 (12.37) | -1.38, 10.54 |
| **PCS** (0-16)  (Rumination) | 6.17 (5.02) | 5.62 (5.01) | 0.55 (3.72) | -0.86, 1.97 | 6.95 (4.13) | 4.58 (4.00) | 2.37 (4.48) | 0.21, 4.53 |
| **PCS** (0-12)  (Magnification) | 4.31 (3.25) | 4.24 (3.7) | 0.07 (2.89) | -1.03, 1.17 | 4.21 (2.9) | 4.0 (3.02) | 0.21 (2.37) | -0.93, 1.35 |
| **PCS** (0-28)  (Helplessness) | 9.86 (6.20) | 8.21 (6.73) | 1.65 (5.63) | -0.49, 3.80 | 10.68 (5.76) | 8.68 (6.78) | 2.00 (6.19) | -0.98, 4.98 |
| **PHQ-9** (0-27) | 11.69 (7.01) | 11.03 (6.38) | 0.66 (5.27) | -1.35, 2.66 | 12.0 (5.50) | 11.84 (5.84) | 0.16 (5.48) | -2.48, 2.80 |
| **BPI** (0-10)  (Interference) | 5.67 (2.50) | 5.28 (2.46) | 0.40 (1.69) | -0.25, 1.04 | 5.95 (2.13) | 5.37 (2.03) | 0.58 (2.50) | -0.62, 1.79 |
| **BPI** (0-10)  (Composite Severity) | 5.48 (1.96) | 5.46 (1.64) | 0.02 (1.41) | -0.53, 0.56 | 5.76 (1.75) | 5.21 (1.78) | 0.55 (2.20) | -0.51, 1.61 |
| **FABQ** (0-42)  (Work) | 16.37 (13.71) | 15.73 (14.66) | 0.63 (11.74) | -3.75, 5.02 | 18.53 (9.6) | 15.84 (9.75) | 2.68 (8.56) | -1.44, 6.81 |
| **FABQ** (0-24)  (Physical Activity) | 13.38 (7.75) | 12.79 (7.71) | 0.59 (7.15) | -2.13, 3.31 | 12.68 (6.68) | 9.79 (5.96) | 2.90 (4.90) | 0.53, 5.26 |
| **SF-36** (0-100)  (Physical Component Score) | 35.35 (15.21) | 39.11 (16.85) | -3.77 (16.28) | -9.96, 2.42 | 29.47 (10.65) | 37.87 (15.24) | -8.4 (12.63) | -14.68, -2.12 |
| **SF-36** (0-100)  (Mental Component Score) | 51.21 (21.73) | 46.69 (21.18) | 4.52 (20.21) | -3.16, 12.21 | 52.09 (18.77) | 48.34 (20.32) | 3.74 (28.58) | -10.47, 17.96 |

**Legend:**

BPI, Brief Pain Inventory; FABQ, Fear Avoidance Belief Questionnaire; GAD-7, General Anxiety Disorder; MOVE group, online interactive programme of mindfulness-based stress reduction and exercise; MCS, Mental component Score; PCS, Pain Catastrophising Scale; PHQ- 9, Patient Health Questionnaire; PDI, Pain Disability Index; PSEQ, Pain Self-Efficacy Questionnaire; SF-36, 36-Item Short Form Survey; SM group, online self-management guide.
