# Supplementary material for: Investigating the long-term impact of a programme of mindfulness combined with exercise delivered online (MOVE) on individuals living with chronic pain-an exploratory one-year follow-up of a feasibility randomised control trial
Source: PLoS One. 2025 Sep 30;20(9):e0323508. doi: 10.1371/journal.pone.0323508 (PMC12483213; doi:10.1371/journal.pone.0323508)
Supplement: S2 File — (DOCX) [file pone.0323508.s002.docx]

**Supplementary File 2 (S2):** Sociodemographic characteristics for participants who returned outcome measures at 1-year and those who did not

| **Participant Characteristic** |  | **Return of Outcome Measures at 1-year follow-up** | | |  |
| --- | --- | --- | --- | --- | --- |
|  | **Yes**  n (%) or mean ± SD | | | **No**  n (%) or mean ± SD | |
|  | **MOVE Group** | | **SM Group** | **MOVE Group** | **SM Group** |
| **Age (years)** | 53.13 ± 12.24 | | 51.56 ± 12.85 | 48.25 ± 11.33 | 48.90 ± 11.79 |
| **Gender (Female)** | 25 (86.2) | | 17 (94.4) | 14 (70) | 24 (82.8) |
| **Pain symptoms with a duration of more than 10 years** | 19 (65.5) | | 10 (55.6) | 12 (60) | 17 (58.6) |
| **Relationship Status** | | | |  |  |
| Married | 19 (65.5) | | 8 (44.4) | 13 (65) | 15 (51.7) |
| In a relationship, not cohabiting | 2 (6.9) | | 1 (5.6) | 1 (5) | 3 (10.3) |
| In a relationship, cohabiting | 0 | | 3 (16.7) | 2 (10) | 2 (6.9) |
| Single | 8 (27.6) | | 6 (33.3) | 4 (20) | 9 (31) |
| **Highest level of Education achieved** | | | |  |  |
| Primary School | 2 (6.9) | | 0 (0) | 2 (10) | 1 (3.4) |
| Secondary School | 5 (17.2) | | 7 (38.9) | 7 (35) | 8 (27.6) |
| Third Level (Undergraduate) | 9 (31) | | 9 (50) | 4 (20) | 7 (24.1) |
| Higher level (post graduate degree) | 9 (31) | | 1 (5.6) | 6 (30) | 9 (31) |
| Other | 4 (13.8) | | 1 (5.6) | 1 (5) | 4 (13.8) |
| **Work Status** | | | |  |  |
| Not working | 13 (44.8) | | 7 (38.9) | 10 (50) | 13 (44.8) |
| Working full time | 2 (6.9) | | 2 (11.1) | 8 (40) | 3 (10.3) |
| Working part-time | 5 (17.2) | | 4 (22.2) | 0 (0) | 6 (20.7) |
| Student | 0 (0) | | 1 (5.6) | 2 (10) | 1 (3.4) |
| Retired | 9 (31) | | 4 (22.2) | 0 (0) | 6 (20.7) |
| **Activity Level** |  | |  |  |  |
| Step-count | 6939 ± 3192 | | 5977 ± 3345 | 7515 ± 3805 | 7195 ± 4053 |
