# Supplementary material for: Investigating the long-term impact of a programme of mindfulness combined with exercise delivered online (MOVE) on individuals living with chronic pain-an exploratory one-year follow-up of a feasibility randomised control trial
Source: PLoS One. 2025 Sep 30;20(9):e0323508. doi: 10.1371/journal.pone.0323508 (PMC12483213; doi:10.1371/journal.pone.0323508)
Supplement: S3 File — (DOCX) [file pone.0323508.s003.docx]

|  | **Supplementary File 3 (S3):** Clinical characteristics for participants who returned outcome measures at 1-year and those who did not | | | | |
| --- | --- | --- | --- | --- | --- |
| **Outcome measure (scoring range)** | | **Return of Outcome Measures at 1-year follow-up** | | | |
|  | | **Yes**  **(n=48)**  Mean ± SD | | **No**  **(n=48)**  Mean ± SD | |
| **Measurement Timepoint** | | **MOVE Group** | **SM Group** | **MOVE Group** | **SM Group** |
| BPI (Interference) (0 - 10) ^¥^ | |  |  |  |  |
| Baseline | | 5.67 ± 2.50 | 5.80 ± 2.08 | 4.87 ± 2.22 | 5.75 ±2.16 |
| 12-week follow-up | | 4.68 ± 2.78 | 5.88 ± 1.91 | 5.09 ± 2.72 | 5.63 ± 1.76 |
| BPI (Composite Severity) (0-10) ^¥^ | |  |  |  |  |
| Baseline | | 5.42 ± 1.95 | 5.71 ± 1.79 | 5.51 ± 1.78 | 5.63 ± 1.88 |
| 12-week follow-up | | 4.94 ± 2.30 | 5.78 ± 1.85 | 5.31 ± 2.49 | 4.58 ± 1.83 |
| PHQ-9 (0-27) ^¥^ | |  |  |  |  |
| Baseline | | 11.69 ± 7.01 | 11.61 ± 5.38 | 13.0 ± 7.46 | 13.38 ± 7.66 |
| 12-week follow-up | | 10.57 ± 7.47 | 10.63 ± 5.37 | 12.31 ± 6.60 | 12.0 ± 6.53 |
| GAD-7 (0–21) ^¥^ | |  |  |  |  |
| Baseline | | 8.97 ± 5.04 | 9.61 ± 5.15 | 9.70 ± 6.33 | 10.66 ± 6.89 |
| 12-week follow-up | | 8.43 ± 6.39 | 8.38 ± 5.37 | 15.43 ± 24.77 | 9.0 ± 6.24 |
| PSEQ (0-60) ^¶^ | |  |  |  |  |
| Baseline | | 29.34 ± 12.31 | 27.72 ± 12.81 | 31.20 ± 11.52 | 26..52 ± 13.77 |
| 12-week follow-up | | 33.64 ± 15.73 | 30.12 ± 12.56 | 29.31 ± 15.01 | 32.80 ± 12.46 |
| PCS total (0-52) ^¥^ | |  |  |  |  |
| Baseline | | 20.43 ± 13.29 | 21.17 ± 12.06 | 19.85 ± 13.48 | 23.82 ± 17.57 |
| 12-week follow-up | | 16.39 ± 15.22 | 18.63 ± 14.46 | 19.38 ± 15.69 | 19.20 ± 16.40 |
| FABQ (Physical Activity) (0-24) ^¥^ | |  |  |  |  |
| Baseline | | 13.38 ± 7.15 | 12.94 ± 6.87 | 10.35 ± 5.62 | 16.0 ± 6.65 |
| 12-week follow-up | | 12.36 ± 6.48 | 9.88 ± 5.84 | 13.54 ± 5.62 | 13.50 ± 5.95 |
| FABQ (Work) (0-42) ^¥^ | |  |  |  |  |
| Baseline | | 16.28 ± 13.94 | 18.22 ± 9.78 | 19.35 ± 12.77 | 17.83 ± 14.35 |
| 12-week follow-up | | 10.0 ± 10.98 | 14.25 ± 13.62 | 21.69 ± 13.34 | 13.00 ± 14.56 |
| PDI (0-70) ^¥^ | |  |  |  |  |
| Baseline | | 37.66 ± 17.32 | 40.39 ± 16.14 | 37.35 ± 19.16 | 39.66 ± 15.47 |
| 12-week follow-up | | 34.21 ± 18.78 | 36.44 ± 15.50 | 37.0 ± 20.84 | 36.50 ± 17.78 |
| SF-36 (PCS) (0-100) ^¶^ | |  |  |  |  |
| Baseline | | 35.35 ± 15.21 | 30.18 ± 10.58 | 35.85 ± 9.40 | 32.22 ±13.79 |
| 12-week-follow-up | | 35.25 ± 13.95 | 36.95 ± 11.01 | 33.68 ± 13.08 | 31.93 ± 9.53 |
| SF-36 (MCS) (0-100) ^¶^ | |  |  |  |  |
| Baseline | | 51.21 ± 21.73 | 51.14 ± 19.02 | 41.03 ± 17.51 | 44.78 ± 23.12 |
| 12-week-follow-up | | 55.43 ± 22.12 | 53.66 ± 21.68 | 43.67 ±21.47 | 49.25 ± 21.87 |

¥ higher scores indicate disimproved symptoms

^¶^ higher scores indicate improved function
